# Triglyceride-glucose index as a marker in cardiovascular diseases; a bibliometric study and visual analysis

**DOI:** 10.1097/MS9.0000000000003019

**Published:** 2025-02-27

**Authors:** Abdulhadi Alotaibi, Abinash Mahapatro, Mohit Mirchandani, Saisree Reddy Adla Jala, Elan Mohanty, Mohammed Dheyaa Marsool Marsool, Herby Jeanty, Pavan Devulapally, Shika M. Jain, Mohammad-Hossein Keivanlou, Pegah Rashidian, Reza Amani-Beni, Maryam Hasanpour, Ehsan Amini-Salehi

**Affiliations:** a Department of Medicine and Surgery, Vision Colleges, Riyadh, Saudi Arabia; b Hi-Tech Medical College and Hospital, Rourkela, Odisha, India; c Montefiore Medical Center Wakefield Campus, New York, USA; d Mission Hospital, Asheville, North Carolina, USA; e Mary Medical Center Apple Valley, California, USA; f Mayo Clinic, Scottsdale, Phoenix, Arizona, USA; g The Brooklyn Hospital Center, Brooklyn, USA; h Main Methodist Hospital, San Antonio, Texas, USA; i MVJ Medical College and Research Hospital, Bengaluru, India; j School of Medicine, Guilan University of Medical Sciences, Rasht, Iran; k Heart Failure Research Center, Cardiovascular Research Institute, Isfahan University of Medical Sciences, Isfahan, Iran

**Keywords:** bibliometric analysis, cardiovascular diseases, data visualization, triglyceride-glucose index

## Abstract

**Objective:**

This study aims to conduct a bibliometric analysis of the triglyceride-glucose (TyG) index in relation to cardiovascular disorders.

**Methods:**

Data for the analysis were extracted from the Web of Science Core Collection database on 13 July 2024. We utilized VOSviewer, CiteSpace, and Biblioshiny tools for the analysis.

**Results:**

The study revealed a marked increase in research outputs on the TyG index in recent years, peaking with 137 publications in 2023. China emerged as the leading contributor, followed by the USA. The Chinese Academy of Medical Sciences and Peking Union Medical College were among the top contributing institutions. Shouling Wu and Shuohua Chen emerged as the leading authors, with the journal Cardiovascular Diabetology publishing the most articles on this topic. Keyword analysis identified “insulin-resistance” as the most frequently occurring term, followed by “risk.” Cluster analysis identified eleven key research areas, including “percutaneous coronary intervention,” “obesity indicators,” “arterial stiffness,” and “heart failure.”

**Conclusion:**

This bibliometric analysis highlights the expanding role of the TyG index in metabolic and cardiovascular research. Key clusters such as percutaneous coronary intervention, obesity indicators, arterial stiffness, heart failure, new-onset hypertension, predicting outcomes, and subclinical coronary artery disease emphasize its wide applicability across diverse clinical settings. The keyword “risk” was the most frequently occurring term, underscoring the importance of the TyG index in cardiovascular risk assessment, alongside its growing use in prognostic applications. These findings reflect the increasing recognition of the TyG index as a pivotal biomarker in cardiovascular medicine and encourage further exploration of its clinical integration.

## Introduction

Cardiovascular diseases (CVDs) remain the leading cause of mortality and disability worldwide, with coronary artery disease (CAD) being particularly prevalent^[1-7]^. CAD is often a precursor to more severe outcomes like heart attacks and heart failure, making early diagnosis and risk assessment critical for improving patient outcomes^[8-11]^.

**Highlights**

- The TyG index is emerging as a key biomarker in cardiovascular research, reflecting its growing role in assessing cardiovascular risk and metabolic health.
- Leading institutions like the Chinese Academy of Medical Sciences and top journals such as Cardiovascular Diabetology are driving significant advancements in TyG-related cardiovascular research.
- Analysis highlights critical research areas, including insulin resistance and cardiovascular risk prediction, underscoring the TyG index’s clinical relevance and potential for personalized treatment strategies.

The triglyceride-glucose (TyG) index, first introduced in the early 2010s, has emerged as a practical and accessible surrogate marker for insulin resistance. It is calculated using the formula: TyG index = ln(fasting triglycerides (mg/dL) × fasting glucose (mg/dL)/2)^[12-15]^. Unlike more complex measures such as the Homeostasis Model Assessment of Insulin Resistance (HOMA-IR), the TyG index is inexpensive, non-invasive, and easily obtainable from standard clinical tests, making it an option for widespread use in both research and clinical practice^[16-20]^. Initial studies linked the TyG index with carotid wall atherosclerosis, but subsequent research expanded its potential as a predictor of other diseases^[13,14]^. Later, more original studies and meta-analyses were published regarding the association between a high TyG index and other metabolic diseases like type 2 diabetes mellitus, metabolic syndrome, non-alcoholic fatty liver disease, hypertension, stroke, heart failure, and diabetic retinopathy (DR)^[15,21-30]^. More recent studies have even associated the TyG index with cardiac valvopathies like aortic stenosis^[31]^.

The cut-off values of TyG index vary across populations and clinical contexts. The optimal cut-off value for the TyG index in adolescents for diagnosing metabolic syndrome was shown to be 8.55 for Mexican Americans, 8.55 for Non-Hispanic Whites, 8.35 for Non-Hispanic Blacks, and 8.45 for Koreans^[32]^. In Brazilian children, cut-offs were 7.9 for boys and 8.1 for girls^[33]^. For predicting CVD and coronary heart disease (CHD) in Iranian populations, a cut-off value of 9.03 has been identified, while values of 8.75 for men and 8.53 for women are used to differentiate prediabetes and diabetes in German adults^[34,35]^.

As research into the TyG index grows, its potential as a tool for early cardiovascular risk assessment is becoming increasingly evident^[15,23,27,36,37]^. However, while the TyG index is gaining traction, its applicability in predicting cardiovascular outcomes is still not fully understood. Exploring this relationship more comprehensively could enhance its utility in clinical practice, offering a simple yet effective means of identifying individuals at high risk of cardiovascular events.

Bibliometric analysis provides a powerful tool for addressing research trends and evolutions. By quantitatively assessing the scientific literature, bibliometrics can uncover patterns in research productivity, impact, and collaboration. This type of analysis not only identifies the most influential studies and researchers but also highlights gaps in the current understanding that could guide future research^[38-41]^.

In this study, we aim to perform a bibliometric analysis of the literature on the TyG index and its association with CVDs. Our goal is to map the research landscape, identifying key contributors, seminal publications, and emerging trends that have shaped the field.

## Methodology

### Data collection

**Figure 1.**
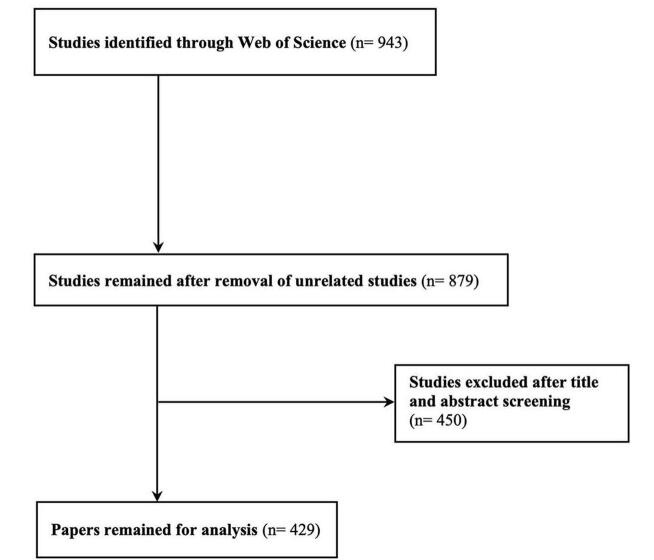
Study selection process.

To obtain the relevant data for our bibliometric analysis on the TyG index in cardiovascular disorders, we utilized the Web of Science Core Collection database on 13 July 2024. This database, known for its extensive repository of over 12 000 scientific articles, provided comprehensive coverage of the research landscape^[42-44]^. Our search strategy, as outlined in Supplementary Digital Content, Table S1 (http://links.lww.com/MS9/A723), employed a combination of keywords including “TyG index,” “Triglyceride-glucose index,” “Cardiovascular Diseases,” “Cardiovascular Abnormalities,” “Heart Disease,” and “Heart Disorder.” The initial search yielded 943 records. After applying refinement criteria to exclude irrelevant studies – such as book chapters, editorials, conference papers, letters, and pre-publication papers – we focused our analysis on 429 articles that directly aligned with our research objectives, as depicted in Fig. 1.

**Figure 2.**
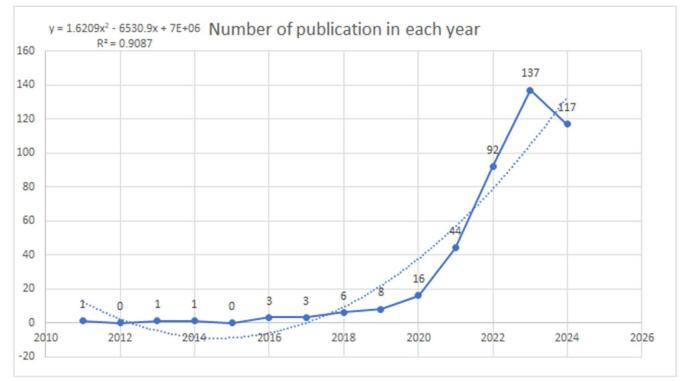
Trends in publication regarding TyG in cardiovascular diseases (dotted line shows the trend line).

### Data analysis

We analyzed the selected articles using three primary tools: VOSviewer (version 1.6.19), CiteSpace (version 6.3 R1), and Biblioshiny (version 4.0), which is part of the Bibliometrix R package. To ensure compatibility with these tools, the data extracted from the Web of Science Core Collection was converted into plain text and CSV formats.

VOSviewer, developed by the Center for Science and Technology Studies at Leiden University, is a robust tool designed for creating and visualizing scientometric networks (accessible at www.vosviewer.com). It excels in generating maps based on network data, allowing for a visual representation of relationships within the scientific literature^[45-47]^. By utilizing VOSviewer, we generated network diagrams that depict co-citation, co-occurrence, citation, and bibliographic coupling among publications, journals, authors, research institutes, countries, and keywords. Text mining methods are employed by VOSviewer, such as part-of-speech tagging algorithms and sentence identification, which are facilitated by the Apache OpenNLP library. While part-of-speech tagging gives each word a part of speech (verb, noun, or adjective), sentence detection divides text data into distinct sentences. This allows for a more thorough study of the text and more precise identification of research themes. In order to facilitate the interpretation of complex bibliometric data, VOSviewer also uses distance-based visualization, in which the distance between nodes indicates the strength of their association^[45-47]^.

CiteSpace is a Java-based application developed by Professor Chaomei Chen at Drexel University (accessible at www.citespace.podia.com). It applies advanced data mining algorithms and in-depth information analysis to construct detailed knowledge maps, revealing the dynamics, structure, and distribution patterns of scientific knowledge. CiteSpace is particularly effective in cluster analysis, which organizes related studies by examining co-citation patterns, thereby highlighting relationships among various research fields. Clusters are assigned labels through log-likelihood ratio tests, which automatically derive significant labels from the key terms found in the articles within each cluster. The quality of these clusters is evaluated using modularity and silhouette scores. Modularity looks at the internal organization of the network, with higher values (closer to 1) indicating clearer, loosely connected sub-networks. Silhouette scores measure the cohesiveness of clusters, with higher scores signifying more uniform and meaningful groupings. Additionally, CiteSpace employs time-slicing, enabling researchers to observe how key concepts evolve over specific periods. Its burst detection algorithm, based on Kleinberg’s approach, identifies rapid increases in citation activity, aiding in the recognition of emerging research trends or breakthroughs^[48-50]^.

Biblioshiny is an online interface for the Bibliometrix R package, created by Massimo Aria and Corrado Cuccurullo (accessible at www.bibliometrix.org). It offers a user-friendly platform for performing bibliometric analyses, featuring a range of analytical tools such as network analysis, descriptive data analysis, and data visualization^[36]^. Moreover, Biblioshiny applies a number of community detection algorithms, including the Walktrap, Louvain, and Multidimensional Scaling algorithms. These algorithms assist in locating clusters within networks and disclose associated study fields or partnerships^[51-54]^.

## Results

### Publication trend

Assessing research trends in a specific field can be done by analyzing the number of publications over time. In the case of the TyG Index and its association with CVDs, there was a notable scarcity of publications from 2011 to 2019, indicating limited research activity during this period. However, starting in 2020, there was a marked increase in publications, indicating a growing interest in the topic. This trend continued, with significant growth observed between 2021 and 2023, culminating in a peak of 137 published articles in 2023. This surge underscores the heightened focus on the potential implications of the TyG index in CVD research. Although there was a slight decline to 117 publications in 2024, this was expected given that the year is still ongoing. These trends reflect an increasing awareness and exploration of the novel possibilities of the TyG index within the cardiovascular field (for a more detailed view, refer to Fig. 2).

**Figure 3.**
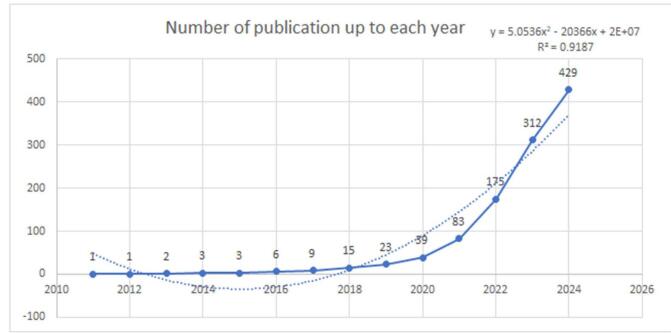
Cumulative publications regarding TyG in cardiovascular diseases (dotted line shows the trend line).

The cumulative production of research articles on the TyG index was noted to have significantly increased between 2011 and 2024. Initially, the growth was gradual, with the number of articles remaining relatively low. By 2019, the cumulative total had been brought to just 23 articles. A notable acceleration in the growth rate was observed from 2020 onwards. This upward trend continued sharply, with the cumulative total increasing to 83 articles by 2021 and surging to 312 articles by 2023. As of 2024, the cumulative production had been brought to 429 articles, reflecting the increasing research interest and activity in this field (for a more detailed view, refer to Fig. 3).

### Countries and institutions

**Figure 4.**
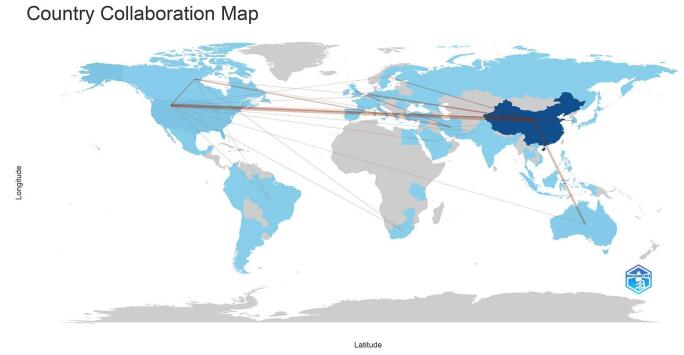
Countries collaboration in the field of TyG in cardiovascular diseases.

**Figure 5.**
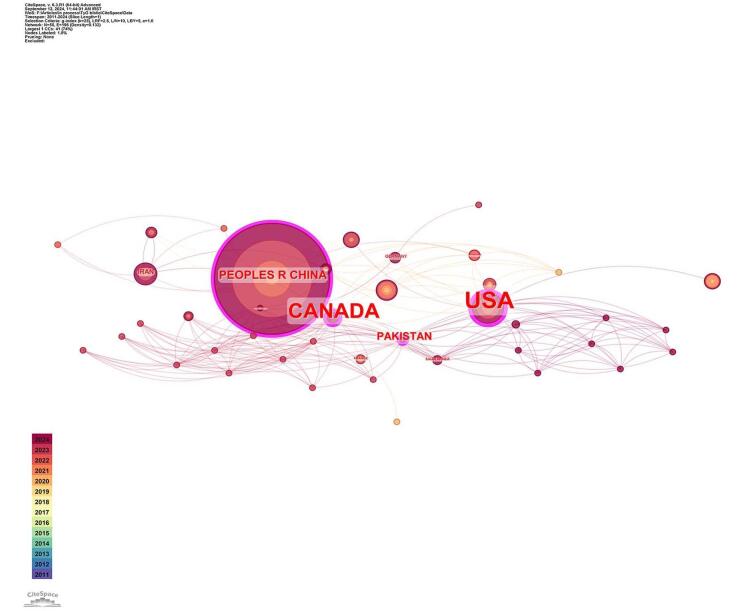
Countries with the high centrality in the field of TyG in cardiovascular diseases.

The analysis of country and institutional contributions revealed significant engagement from various nations in the field of TyG in CVDs. As illustrated in Fig. 4, the collaboration among these countries was mapped. A total of 55 countries were identified as contributors to this research area. China was identified as the leading contributor with the highest number of publications (*n* = 324), followed by the USA (*n* = 27) and Turkey (*n* = 27). Other notable contributors included South Korea (*n* = 22), Iran (*n* = 14), Australia (*n* = 9), Taiwan (*n* = 9), Brazil (*n* = 7), and Italy (*n* = 6). Table 1 presents the top 10 countries along with their respective number of publications and centrality values. The USA held the highest centrality value (0.32), indicating its significant influence in the field, followed by China (0.17) and Canada (0.28). Fig. 5 highlights the countries with high centrality, showcasing their roles in advancing research on TyG in CVD.

**Table 1:** Top 10 countries and institutions in the field of TyG in cardiovascular diseases

Rank	Country	Contribution	Centrality	Institution	Contribution	Centrality
1	China	324	0.17	Chinese Academy of Medical Sciences	50	0.19
2	USA	27	0.32	Peking Union Medical College	39	0.09
3	Turkey	27	0.00	Capital Medical University	35	0.10
4	South Korea	22	0.00	Fu Wai Hospital – CAMS	30	0.05
5	Iran	14	0.09	Nanchan University	17	0.05
6	Australia	9	0.01	Nanjing Medical University	17	0.03
7	Taiwan	9	0.00	North China University of Science and Technology	14	0.05
8	Brazil	7	0.00	Zhengzhou University	14	0.04
9	Italy	6	0.02	Chinese People’s Liberation Army General Hospital	13	0.03
10	England	5	0.00	Sun Yat-sen University	13	0.06

A total of 519 institutions have participated in research on TyG and CVDs. The Chinese Academy of Medical Sciences (*n* = 50) had established itself as the foremost institution in terms of publication count, followed closely by Peking Union Medical College (*n* = 39) and Capital Medical University (*n* = 35). Additionally, Fu Wai Hospital – CAMS (*n* = 30) and Nanchang University (*n* = 17) also played significant roles in advancing research, highlighting the collaborative efforts within the academic community (for a more detailed view, refer to Table 1).

**Figure 6.**
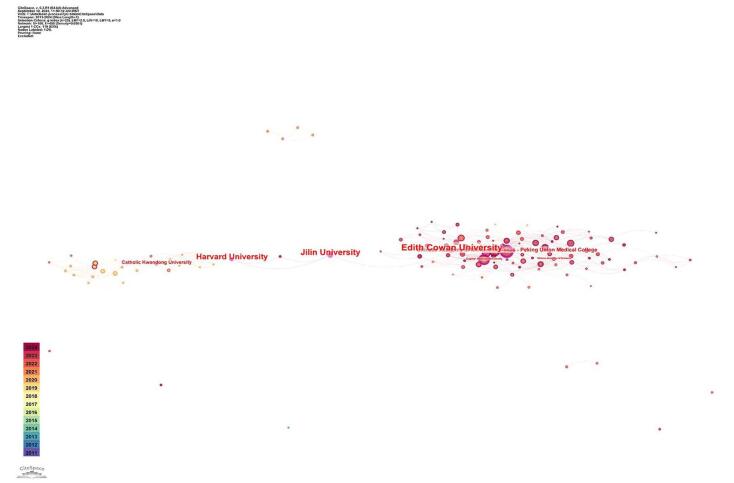
Institutions with the high centrality in the field of TyG in cardiovascular diseases.

Regarding centrality, Edith Cowan University had emerged as the leading institution, with a centrality score of 0.32. Jilin University closely followed, with a centrality score of 0.30. Harvard University also stood out, having recorded a centrality score of 0.28. The Chinese Academy of Medical Sciences had a centrality score of 0.19. Catholic Kwandong University achieved a centrality score of 0.17 (for a more detailed view, refer to Table 1 and Fig. 6).

### Journals and co-cited journals

**Figure 7.**
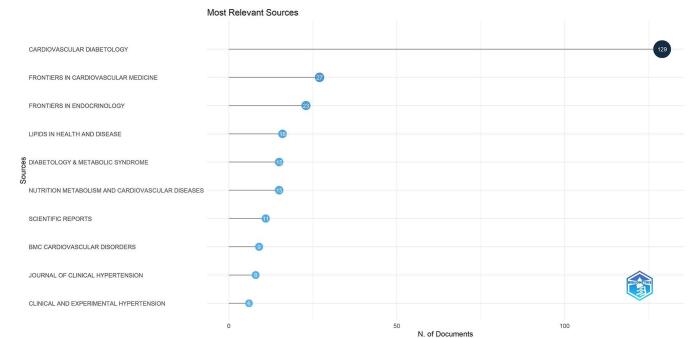
The most relevant journals in the field of TyG index in cardiovascular disorders.

A total of 132 journals were identified in the analysis. As shown in Fig. 7, the journal “Cardiovascular Diabetology” stood out with the highest number of relevant documents, totaling 129. It was followed by “Frontiers in Cardiovascular Medicine” and “Frontiers in Endocrinology,” which had 27 and 23 documents, respectively. Other significant contributions included “Lipids in Health and Disease” with 16 documents, “Diabetology & Metabolic Syndrome” with 15 documents, and “Nutrition Metabolism and Cardiovascular Diseases” also with 15 documents. These journals provided a substantial portion of the literature pertinent to our study on the TyG index. Additionally, “Scientific Reports” contributed 11 documents, while “BMC Cardiovascular Disorders” and “Journal of Clinical Hypertension” contributed 9 and 8 documents, respectively. “Clinical and Experimental Hypertension” completed the list with 6 documents.

**Figure 8.**
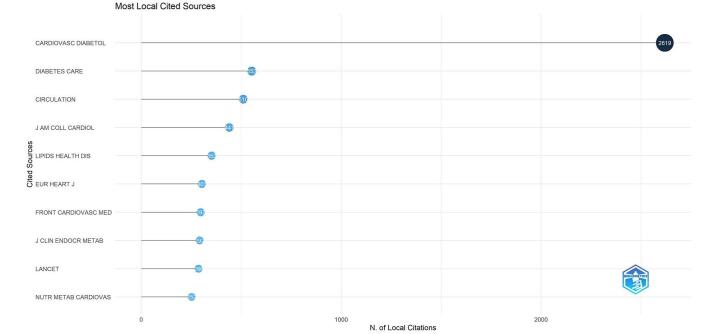
The most cited journals in the field of TyG index in cardiovascular disorders.

The analysis of the most locally cited sources revealed that “Cardiovascular Diabetology” was the most frequently cited, with 2619 citations. It was followed by “Diabetes Care,” which received 510 citations. “Circulation” was also highly cited, with 441 citations. Other notable sources included the “Journal of the American College of Cardiology (J Am Coll Cardiol),” cited 352 times, and “Lipids in Health and Disease,” which garnered 303 citations. The “European Heart Journal (Eur Heart J)” and “Frontiers in Cardiovascular Medicine” were cited 297 and 292 times, respectively. Additionally, both the “Journal of Clinical Endocrinology & Metabolism (J Clin Endocr Metab)” and “The Lancet” received 286 citations each. Finally, “Nutrition Metabolism and Cardiovascular Diseases” had 252 citations (for a more detailed view, refer to Fig. 8).

**Figure 9.**
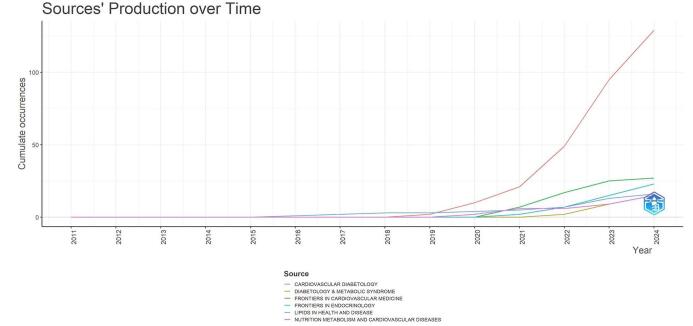
Journals’ productions over time in the field of TyG-index in cardiovascular disorders.

The examination of publishing patterns over time for leading sources in CVD research related to the TyG index revealed notable shifts in scholarly production. Fig. 9 illustrates a significant and rapid increase in publications for the journal Cardiovascular Diabetology, with a particularly steep rise evident from 2020 onward. Other journals experienced slower but steady growth. Diabetology & Metabolic Syndrome and Frontiers in Cardiovascular Medicine demonstrated considerable expansion, particularly starting in 2020. The trajectories of Frontiers in Endocrinology and Lipids in Health and Disease were of particular interest, as they displayed consistent upward trends that began to diverge in 2019.

### Authors and co-cited authors

**Figure 10.**
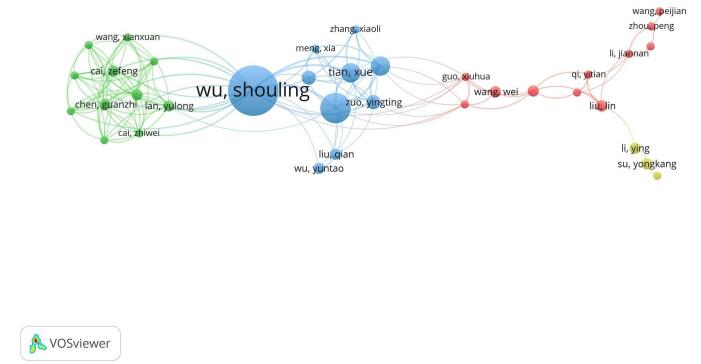
Network visualization of the top authors in the field of TyG index in cardiovascular disorders.

Our bibliometric analysis identified the leading authors who contributed to the study of the TyG index in relation to cardiovascular disorders (for a more detailed view, refer to Table 2). A total of 2589 authors were recognized. Shouling Wu emerged as the most prolific author with 18 publications. Shuohua Chen followed with 11 publications. Other notable contributors included Xue Tian and Anxin Wang, each with seven publications. Yingting Zuo also made significant contributions, having authored five documents (for a more detailed view, refer to Fig. 10).

**Table 2:** The most cited and co-cited authors regarding the TyG index in cardiovascular disorders

Rank	Author	Citations	Documents	Author	Co-citations
1	Wu, Shouling	424	18	Guerrero-Romero, Fernando	248
2	Chen, Shuohua	328	11	Simental-Mendia, Luis E	203
3	Tian, Xue	238	7	Ormazabal, V	125
4	Wang, Anxin	238	7	Won, Ki-Bum	119
5	Zheng, Jingang	35	6	Vasques, Ana Carolina Junqueira	117
6	Wang, Yongjun	125	5	Jin, Jing-Lu	102
7	Zuo, Yingting	229	5	Park, Kahui	99
8	Gao, Yanxiang	32	5	Wang, I	97
9	Li,Yike	32	5	Da Silva, Alessandra	92
10	Xie, Enmin	32	5	Zhao, Qi	87

**Figure 11.**
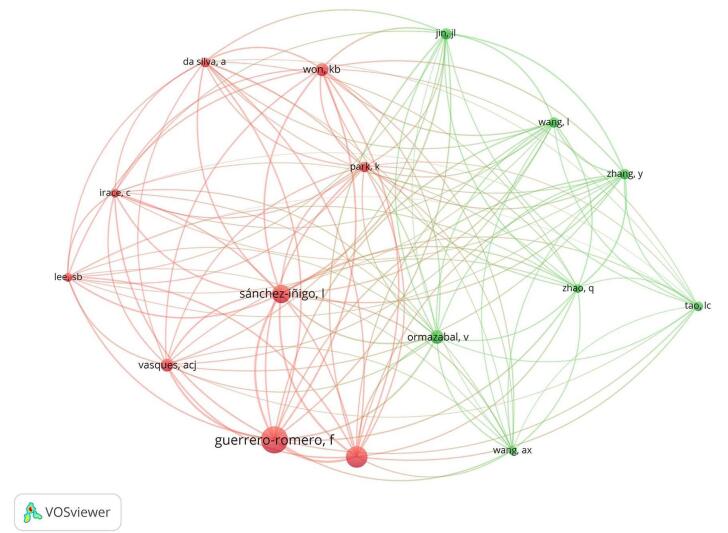
Overlay visualization of the top co-cited authors cited in the field of TyG index in cardiovascular disorders.

Our analysis also identified the most co-cited authors (for a more detailed view, refer to Table 2 and Fig. 11). Guerrero-Romero, F. was distinguished as the most co-cited author, with a total of 248 citations. Sánchez-Ñigo, L., and Simental-Mendia, L. E. also ranked prominently, with 171 and 203 citations, respectively. Additionally, Won, KB, Jin, and JL were recognized as key figures due to their significant number of citations and strong link strengths.

### Top cited references

Table 3 presents the top 10 most cited articles within the field, highlighting their significant contributions to the understanding of the TyG index and its implications on cardiovascular health. The article titled “TyG index performs better than HOMA in a Brazilian population: a hyperglycemic clamp validated study,” published in 2011, held the highest citation count with 388 citations. This foundational study established the TyG index as a more effective measure compared to the Homeostasis Model Assessment (HOMA) in assessing insulin resistance. Following closely, the 2016 article “The TyG index may predict the development of cardiovascular events” garnered 293 citations, emphasizing the predictive value of the TyG index in cardiovascular risk assessment. In 2022, the review “Triglyceride-glucose index as a marker in CVDs: landscape and limitations” accumulated 194 citations, providing a comprehensive overview of the TyG index’s role and its limitations in CVD research. Other notable studies included “Markers of insulin resistance and carotid atherosclerosis” (2013) with 165 citations, which compared various indices for assessing insulin resistance, and a meta-analysis published in 2021 that linked the TyG index to the incidence of atherosclerotic CVDs, accumulating 149 citations. The remaining articles, ranging from 121 to 146 citations, further explored the association of the TyG index with hypertension and CAD, underscoring its relevance in ongoing cardiovascular research.

**Table 3:** Top 10 cited references in the field of TyG-index in cardiovascular disorders

Rank	Title of the most cited paper	Year	DOI	Total citations
1	TyG index performs better than HOMA in a Brazilian population: a hyperglycemic clamp validated study	2011	10.1016/j.diabres.2011.05.030	388
2	The TyG index may predict the development of cardiovascular events	2016	10.1111/eci.12583	293
3	Triglyceride-glucose index as a marker in cardiovascular diseases: landscape and limitations	2022	10.1186/s12933-022-01511-x	194
4	Markers of insulin resistance and carotid atherosclerosis. A comparison of the homeostasis model assessment and triglyceride glucose index	2013	10.1111/ijcp.12124	165
5	Triglyceride-glucose index and the incidence of atherosclerotic cardiovascular diseases: a meta-analysis of cohort studies	2021	10.1186/s12933-021-01268-9	149
6	High triglyceride-glucose index is associated with poor prognosis in patients with acute ST-elevation myocardial infarction after percutaneous coronary intervention	2019	10.1186/s12933-019-0957-3	146
7	Triglyceride and glucose (TyG) index as a predictor of incident hypertension: a 9-year longitudinal population-based stud	2017	10.1186/s12944-017-0562-y	138
8	Triglyceride and glucose (TyG) index as a predictor of incident hypertension: a 9-year longitudinal population-based study	2020	10.1186/s12916-020-01824-2	127
9	Triglyceride and glucose (TyG) index as a predictor of incident hypertension: a 9-year longitudinal population-based study	2018	10.21037/jtd.2018.10.79	124
10	Triglyceride-glucose index is associated with symptomatic coronary artery disease in patients in secondary care	2019	10.1186/s12933-019-0893-2	121

### Keyword trends

**Figure 12.**
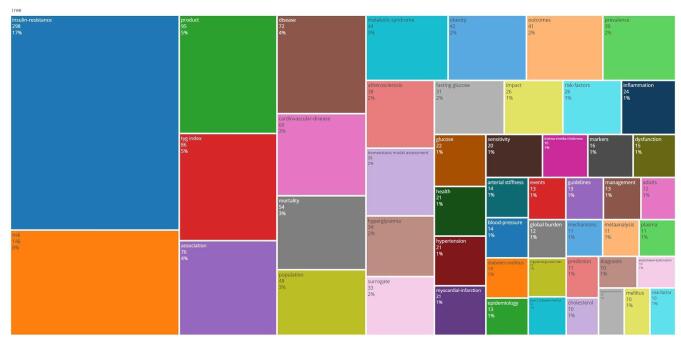
Tree map of the most used keywords in the field of TyG-index in cardiovascular disorders.

Our analysis of keyword trends revealed that the most significant keyword was “insulin-resistance,” with 298 occurrences. The second most frequent keyword was “risk,” appearing 146 times. “Product” and “TyG index” were also notable, with 95 and 86 occurrences, respectively. Additionally, “cardiovascular-disease” was found 60 times. Other important keywords included “mortality” (54 occurrences), “population” (48 occurrences), and “metabolic syndrome” (44 occurrences) (for a more detailed view, refer to Fig. 12).

**Figure 13.**
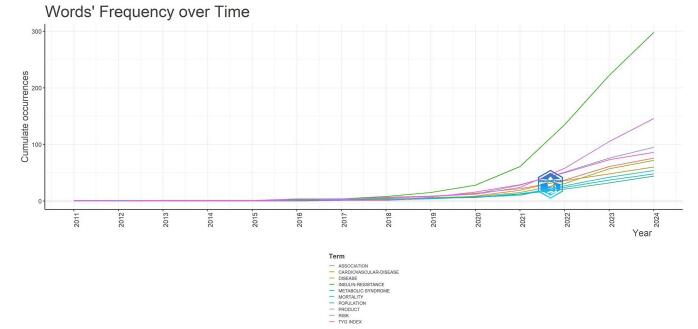
Keyword trends and their frequency over time in the field of TyG in CVD.

Regarding word frequency over time, “insulin-resistance” has shown a significant increase, especially from 2018 onwards. By 2024, it had reached over 298 cumulative occurrences. The term “risk” also exhibited a notable upward trend, particularly after 2018, with approximately 146 occurrences by 2024. This trend indicated a heightened focus on risk assessment in the context of the TyG index and cardiovascular health. Key terms such as “cardiovascular-disease” and “metabolic syndrome” displayed steady growth over the years. “Cardiovascular-disease” began to rise around 2017 and continued to increase, reaching over 60 occurrences by 2024. Similarly, “metabolic syndrome” demonstrated a consistent upward trajectory, underscoring its relevance in this research area. The keywords “population,” “mortality,” “product,” and “TyG index” also showed a gradual increase over time, with a more pronounced rise observed from 2020 onwards (for a more detailed view, refer to Fig. 13).

### Hotspots and cluster analysis

**Figure 14.**
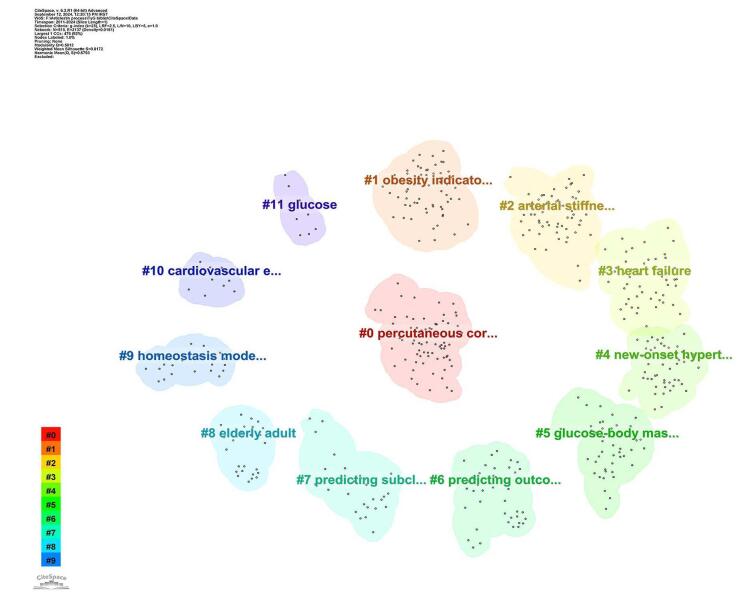
Cluster analysis of the topics related to TyG index in cardiovascular disorders.

Through cluster analysis, 11 main clusters were identified, each representing significant areas of research focus. The key clusters included percutaneous coronary intervention (#0), obesity indicator (#1), arterial stiffness (#2), heart failure (#3), new-onset hypertension (#4), glucose-body mass index (#5), predicting outcome (#6), predicting subclinical CAD (#7), elderly adult (#8), homeostasis model assessment (#9), cardiovascular event (#10), and glucose (#11). These clusters highlight diverse research areas, including cardiovascular interventions, metabolic indicators, and predictive modeling in clinical settings (for a more detailed view, refer to Fig. 14).

**Figure 15.**
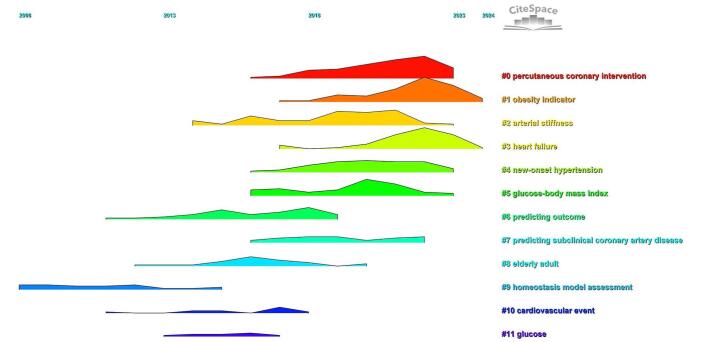
Time trend analysis of the topics related to TyG index in cardiovascular disorders.

The time trend analysis further demonstrated the evolution of these research clusters, revealing shifts in research priorities over time. Recent years have seen an increased emphasis on emerging clusters such as “percutaneous coronary intervention,” “obesity indicator,” and “heart failure” (for a more detailed view, refer to Fig. 15).

## Discussion

TyG index has become increasingly important in CVD research due to its ability to predict and evaluate disease risk and progression^[16]^. Further research has established the TyG index’s predictive power in CVD contexts. For example, a study by Sánchez-Íñigo *et al*, using data from the vascular metabolic CUN cohort, demonstrated a positive association between the TyG index and CVD events, including CHD, cerebrovascular disease, and peripheral arterial disease. This study was particularly notable for its large sample size and long follow-up period of 10 years^[55]^.

The TyG index has demonstrated a substantial link with stable CAD for particular CVDs. A higher TyG index is consistently associated with poor outcomes for patients with CAD, according to clinical data. One nested case-control research, for example, indicated that, after controlling for confounding variables, a higher TyG index was linked to an increased risk of major adverse cardiovascular and cerebral events (MACCEs) in 1282 patients with type 2 diabetes mellitus (T2DM) and new-onset stable CAD^[56]^. The TyG index’s predictive significance in patients with stable CAD was validated by Jin *et al* in another study^[57]^. Asymptomatic individuals with a high risk of atherosclerosis can also be effectively recognized using the TyG index. In a study by Lee *et al*, a greater TyG index was linked to an increased risk of coronary artery stenosis, establishing it as an independent risk factor for CAD^[58]^. Higher TyG index levels were associated with both the number and severity of narrowed coronary arteries, according to research conducted in Vietnam by Tai *et al*^[59]^.

Numerous meta-analyses have examined the association between the TyG index and various health outcomes. A recent meta-analysis, which analyzed ten observational studies involving 13 716 patients with type 2 diabetes, revealed that a higher TyG index was linked to a 2.34 times greater risk of developing DR compared to those with a lower TyG index^[22]^. Furthermore, another meta-analysis found that individuals in the highest TyG index category at baseline were independently associated with an increased risk of stroke when compared to those in the lowest category^[60]^. Previous research also established significant links between a high TyG index and conditions such as type 2 diabetes mellitus, atrial fibrillation, contrast-induced nephropathy, and non-alcoholic fatty liver disease^[26,61-63]^.

This bibliometric study employed data visualization and scientometric analysis to investigate the current landscape of TyG index applications in CVD. We identified and highlighted the leading countries, their collaborative networks, and key institutions and authors in this field. Furthermore, we pinpointed major research hot spots and uncovered various areas within the field.

Our study found that the number of publications in the field has been steadily increasing. This growing body of work points to a significant trend in both medical research and clinical practice. The limited number of publications between 2011 and 2018 indicates that this was a relatively unexplored area during this period. However, the noticeable increase in publications starting from 2019 reflects a growing recognition of the potential implications of the TyG index in cardiovascular research. This surge might be due to several factors; for example, the TyG index has gained recognition as a reliable and cost-effective marker for insulin resistance, which is a critical risk factor for CVDs^[34,64-67]^. Studies validating the TyG index against other established markers have contributed to its growing acceptance and use in research. In addition, Advances in research methodologies, including improved data analytics, machine learning, and big data, have enabled more comprehensive and precise studies on the TyG index^[57]^. The relationship between baseline TyG index and cardiovascular events in the general population has been the focus of numerous prior investigations^[34,55,67-71]^. A higher baseline TyG index was associated with an 84% greater risk of coronary heart disease and a 61% increased risk of CVD, according to the Tehran Lipid and Glucose Study, which included a 16-year follow-up^[34]^.

The global interest in the TyG index and its cardiovascular consequences is evident from the geographical distribution of study contributions. China is at the top of the list. China’s dominant role in studying the TyG index and CVD may be credited to several linked variables, which demonstrate its strategic emphasis on developing healthcare research and tackling public health issues. The Chinese government’s significant allocation of funds for scientific research infrastructure has established a strong basis for the country’s prominent position. Prestigious research organizations and colleges, such as the Chinese Academy of Medical Sciences and Peking Union Medical College, have received substantial financing, which has allowed them to carry out comprehensive research. These institutes possess cutting-edge infrastructure and have the ability to utilize extensive datasets, enabling thorough research on new biomarkers such as the TyG index^[72]^. Moreover, China is confronted with a substantial public health challenge as a result of the widespread occurrence of metabolic diseases, such as T2DM, obesity, and CVDs^[73-75]^. The rising prevalence of these diseases has created a need to find and verify efficient biomarkers for early identification, risk evaluation, and treatment. The TyG index has gained significant attention in the Chinese medical research community due to its cost-effectiveness and dependability as a marker for insulin resistance^[76]^. The significant contributions from the United States, South Korea, and other nations exemplify the universal acknowledgment of the significance of the TyG index in cardiovascular health. The USA and Canada have high centrality scores, indicating that both nations have crucial responsibilities in promoting international cooperation and advancing research in this field.

By analyzing journals and co-cited journals, it has been determined that “Cardiovascular Diabetology” is the most prominent source of papers on the TyG index and CVDs. The abundance of relevant information and local citations highlight its pivotal significance in sharing crucial discoveries in this field of research. The existence of journals such as “Frontiers in Cardiovascular Medicine,” “Frontiers in Endocrinology,” and “Lipids in Health and Disease” underscores the multidisciplinary nature of this investigation, connecting the fields of cardiology, endocrinology, and lipid metabolism. The citation trends are indicative of the journals’ influence and impact on the scientific community. The exponential increase in articles in the field of “Cardiovascular Diabetology” and the continuous contributions from other influential journals indicate that research on the TyG index is acquiring substantial momentum. The substantial number of citations received by publications such as “Diabetes Care” and “Circulation” signifies their crucial significance in offering fundamental information and context for research on the TyG index and its impact on cardiovascular health.

The identification of leading authors in the field highlights the key contributors to research on the TyG index and CVDs. Authors like Shouling Wu and Shuohua Chen, with their substantial number of publications and citations, have significantly advanced our understanding of the TyG index. The examination of co-cited authors highlights the key studies that support contemporary research. Authors such as Fernando Guerrero-Romero, and Luis E Simental-Mendia have been frequently cited, indicating their pivotal contributions to the field. The co-citation patterns suggest a well-connected research community with a shared foundation of key studies, facilitating the ongoing exploration of the TyG index in cardiovascular health.

The study conducted by Vasques *et al* is considered the most prominent in this field. It focused on evaluating the performance of the TyG index in detecting insulin resistance (IR) compared to the HOMA-IR^[12]^. The study sample consisted of 82 Brazilian patients. This study found that the TyG index had a stronger connection with measures of body fat, metabolism, and early-stage heart disease compared to the HOMA-IR index. The TyG index, derived from fasting levels of triglycerides and glucose, had a notable association with measures such as visceral adipose tissue and HDL cholesterol, indicating its reliability and accessibility as a tool for clinical treatment. The modest concurrence with the hyperglycemic clamp, which is considered the most accurate method for measuring insulin resistance, highlights its potential usefulness in many clinical environments, especially when advanced testing is not possible. In the second most influential study, Sánchez-Íñigo *et al* investigated the ability of the TyG index to predict CVD events in a group of 5014 individuals^[55]^. The patients were followed for a median time of 10 years. Their research revealed that elevated TyG index values were strongly linked to a greater likelihood of developing CVD, regardless of conventional risk variables. The study employed Cox proportional-hazard models and discovered that the inclusion of the TyG index in the Framingham risk model enhanced its ability to predict outcomes. This indicates that the TyG index can function as a straightforward and cost-efficient tool for early detection of persons with a high risk of CVD, which might possibly improve preventive measures and enhance patient outcomes. The third most influential study was by Tao *et al*, which conducted an in-depth review of the TyG index as a substitute indicator for insulin resistance and its correlation with CVDs^[16]^. The study emphasized strong evidence establishing a connection between the TyG index and the occurrence and outlook of different forms of CVD. Although the TyG index shows promise in its use, the research also highlighted the limits and challenges associated with employing it as a predictive marker. These factors encompass variations in threshold levels among various groups and the requirement for more research to clarify the underlying mechanisms.

The three studies collectively emphasize the increasing acknowledgment of the TyG index as a viable tool in both clinical and epidemiological contexts. The initial research conducted by Vasques *et al* showcases the index’s efficacy and dependability in evaluating insulin resistance, a crucial aspect in the management of metabolic illnesses. The research conducted by Sánchez-Íñigo *et al* expands the applicability of the TyG index in forecasting cardiovascular events, demonstrating its promise in preventive healthcare. The review conducted by Tao *et al* offers a comprehensive outlook on the current situation and emphasizes the importance of additional verification. It underscores the delicate equilibrium between the potential usefulness of the subject matter and the imperative for carefulness and uniformity.

The high frequency of the keyword “insulin-resistance” in studies on the TyG index and CVDs highlights its crucial significance in the underlying mechanisms of both metabolic and cardiovascular disorders. Insulin resistance is a state in which the cells of the body become less receptive to the effects of insulin, resulting in higher levels of glucose in the bloodstream^[77]^. The metabolic dysfunction described here is a fundamental aspect of both T2DM and metabolic syndrome, both of which are significant contributors to the development of CVDs^[78]^. And since the TyG index is a composite measure of fasting triglycerides and glucose levels, it is widely acknowledged as a reliable surrogate marker for insulin resistance. As a result, researchers often investigate the correlation between the TyG index and insulin resistance to determine its ability to predict cardiovascular outcomes. The other most commonly used keyword, “risk,” is as widespread as it encompasses the main focus of cardiovascular research, which seeks to discover and reduce variables that lead to cardiovascular illnesses. The TyG index is receiving recognition as a possible indicator for evaluating the likelihood of acquiring cardiovascular problems^[55,57,69,79]^. Research often examines the association between high TyG index levels and heightened susceptibility to events including heart attacks, strokes, and other cardiovascular problems. Also, the keyword “Metabolic syndrome” is commonly mentioned in keyword trends since it is closely linked to both insulin resistance and cardiovascular disorders. Metabolic syndrome is a collection of interconnected diseases, such as higher blood pressure, high blood sugar, excessive abdominal fat, and abnormal cholesterol levels, that coexist and amplify the likelihood of developing heart disease, stroke, and diabetes^[80,81]^. The TyG index is highly significant in the context of metabolic syndrome due to its incorporation of triglyceride and glucose levels, which are fundamental elements of this illness^[82,83]^. The research frequently centers around the utilization of the TyG index as a diagnostic and prognostic indicator for metabolic syndrome, as well as its ability to identify patients with a high likelihood of developing cardiovascular issues. The convergence of metabolic syndrome, CVD, and insulin resistance renders it a pivotal term in TyG index investigation. Finally, the keyword “mortality” is crucial in TyG index research since it specifically pertains to the primary clinical outcome of interest: the rates of death related to cardiovascular illnesses. Gaining a comprehensive understanding of the relationship between the TyG index and mortality rates can offer valuable insights into its predictive significance^[36,66,83,84]^. Studies frequently investigate the correlation between elevated TyG index readings and higher rates of both all-cause mortality and cardiovascular-specific death.

The TyG index has been significantly linked to various metabolic disorders; however, a recent umbrella review has provided the limitations which should be addressed in future studies. This review identified a notable connection between the TyG index and several conditions, including non-alcoholic fatty liver disease, contrast-induced nephropathy, DR, type 2 diabetes mellitus, coronary artery calcification, chronic kidney disease, and atrial fibrillation. Despite these findings, the epidemiological strength, assessed using GRADE criteria, was deemed low for most outcomes^[15]^.

In clinical settings, the TyG index, derived from fasting triglycerides and fasting glucose, has proven to be a practical and cost-effective marker for identifying insulin resistance and assessing populations at high risk for diabetes^[85,86]^. It has been reported to outperform the HOMA-IR index in predicting atherosclerosis-related conditions, such as carotid atherosclerosis and coronary artery calcification progression^[13,87]^. Unlike the HOMA-IR, which requires insulin measurements that are often costly and unavailable in many resource-limited settings, the TyG index offers a more accessible alternative. Furthermore, because it does not rely on insulin levels, it is found to be particularly useful for diabetic patients receiving insulin therapy^[16]^. This simplicity and broad applicability made the TyG index a valuable tool for evaluating insulin resistance in clinical and research contexts, especially in developing regions.

However, the clinical use of the TyG index in CVDs has certain limitations. Its reliability is shown to be influenced by conditions such as hyperlipidemia and diabetes, which may confound its association with cardiovascular events^[16]^. For example, studies by Laura *et al* and Cho *et al* reported no significant association between the TyG index and CVD outcomes in patients with well-controlled diabetes or hypertension, likely due to the effects of medication use and healthier lifestyle adaptations^[55,65]^. Additionally, many studies lack detailed information about factors such as medication regimens, physical activity, alcohol consumption, and family history, which can affect the findings^[16]^.

Another challenge identified is whether the TyG index provided additional predictive value beyond its individual components, TG and FG. Most studies relied on a single baseline measurement, overlooking temporal variations that could affect its accuracy, particularly in dynamic conditions like acute myocardial infarction, where stress-induced hyperglycemia could alter the index. Cui *et al* demonstrated that a cumulative TyG index, calculated over multiple time points, was a stronger predictor of CVD outcomes, with an adjusted hazard ratio of 1.39 for higher quartiles^[65]^. This finding highlighted the need for longitudinal assessments to improve the reliability of the TyG index as a predictive tool.

Another key concern is its variable predictive value across different populations. Studies suggest that the effectiveness of the TyG index can differ significantly among ethnic groups and in populations with specific health conditions. These disparities highlight the need for further research to validate its applicability in diverse demographic and clinical contexts^[32,88-91]^. Another limitation is the lack of standardization in its use. Currently, there is no universally agreed-upon cut-off value for the TyG index, making it challenging to interpret and compare results across studies^[23,92,93]^.

Additionally, while the TyG index is associated with an increased risk of cardiovascular outcomes, it lacks sensitivity or specificity in some population^[94,95]^. This limitation may reduce its utility in cases where more precise diagnostic markers are required to guide treatment decisions.

Another limitation is the presence of consistent biases across the existing literature. Population bias is evident, as certain populations, such as those in African countries, remain underrepresented, limiting the generalizability of findings. Publication bias may also lead to an overrepresentation of studies with positive findings, potentially skewing perceptions of the TyG index’s utility. The dominance of observational and retrospective study designs further restricts causal inference. Additionally, variability in methodologies, such as reliance on single-point measurements and the absence of standardized cut-off values, complicates cross-study comparisons. Confounding factors, including lifestyle, medication use, and co-morbid conditions, may influence reported associations. Addressing these issues through robust, diverse, and longitudinal studies is essential to enhance the clinical applicability of the TyG index.

In summary, our bibliometric study highlighted the TyG index as an emerging biomarker in CVDs, alongside more traditional markers like LDL cholesterol, blood pressure, and glucose levels^[96,97]^. TyG index is a cost-effective and reliable surrogate for insulin resistance, which plays a crucial role in the pathophysiology of CVDs^[98,99]^. Comparing our findings with existing bibliometric studies, we observed that while traditional risk factors remain central in cardiovascular research, newer markers such as the TyG index are gaining traction. For instance, previous bibliometric studies have focused on metabolic and environmental risk factors, including lipid abnormalities, hypertension, and lifestyle-related behaviors like sedentary behavior^[100,101]^. These studies are consistent with our findings, as insulin resistance and metabolic syndrome are recognized as key contributors to cardiovascular risk^[99,102,103]^. What distinguished the TyG index from traditional markers is its simplicity and broad applicability, particularly in clinical settings where more complex assessments like the HOMA-IR may not be feasible^[104,105]^. Furthermore, our study revealed that the TyG index is increasingly used as a risk factor for CVDs, a trend that aligned with evolving research interests in using metabolic markers to predict CVD outcomes^[15,106]^. This growing body of evidence supports the integration of the TyG index into clinical practice as a straightforward yet effective tool for cardiovascular risk stratification. By placing our findings within the context of these broader bibliometric studies, we illustrated that the TyG index is not only gaining attention but also contributing meaningfully to the expanding landscape of cardiovascular research.

### Limitations

Despite the broad nature of our bibliometric study, it is important to acknowledge some limitations. Firstly, our analysis was limited to articles listed in the Web of Science Core Collection. While this is a respected database for high-quality academic publications, it does not encompass all relevant literature. Studies published in other databases such as Scopus, and PubMed were not included, potentially resulting in the omission of some contributions. Additionally, non-indexed journals, which may contain regionally relevant or innovative studies, were also left out. This limitation could introduce bias in our findings, as the exclusion of studies from certain databases may lead to an underrepresentation of key regions or alternative methodologies in TyG index research.

Second, the scope of our study was limited to articles published in English, potentially excluding significant research published in other languages. This language bias may particularly affect the representation of non-English-speaking regions that are engaged in cardiovascular research, such as parts of Asia, Latin America, and Europe. Including non-English studies in future bibliometric analyses would improve the global perspective and reveal diverse scientific contributions that are currently underrepresented.

Third, the bibliometric tools utilized in this study – VOSviewer, CiteSpace, and Biblioshiny – rely on citation data. While these tools are helpful for mapping research trends, it’s important to note that citation counts may not always accurately reflect research quality. Various factors such as the popularity of specific journals, author partnerships, or trendy research topics can influence citation counts, potentially leading to an incomplete or biased representation of scientific impact. This means that studies that are highly relevant but published in less-visible journals may not be accurately represented.

Fourth, while our study identifies key contributors, influential publications, and emerging trends in the TyG index and cardiovascular research, it does not assess the qualitative content or scientific rigor of individual studies. For instance, there is no detailed evaluation of study designs, methodologies, or outcomes, which could provide a more comprehensive understanding of how the TyG index is being applied across different clinical and research settings. Future research should incorporate qualitative assessments to evaluate the methodological robustness and clinical relevance of the literature, potentially offering deeper insights into how the TyG index is utilized and interpreted. Incorporating mixed methods that combine bibliometric analysis with systematic reviews, meta-analyses, or even umbrella reviews could offer a more comprehensive perspective. Such an approach would not only map the research landscape quantitatively but also qualitatively assess the quality and clinical relevance of the studies on the TyG index. By systematically evaluating the methodological robustness of key studies, future research could provide deeper insights into how the TyG index is applied across different populations, disease settings, and clinical practices. For instance, qualitative assessments could help identify gaps in study designs, potential biases, and limitations in existing research. This would offer a better understanding of the TyG index’s utility and contribute to refining its role as a marker of cardiovascular risk.

Lastly, the field of cardiovascular research and biomarkers like the TyG index is rapidly evolving. As new research emerges, particularly with advances in technologies like machine learning, genomics, and personalized medicine, future studies may rapidly alter the trends observed in our bibliometric analysis. Therefore, it is important to acknowledge that this study provides a snapshot of the field as of 2024, and the results may need frequent updating to reflect new developments in the literature.

Future bibliometric studies should incorporate data from multiple databases, including Scopus, PubMed, and Embase, to provide a more comprehensive analysis. This would ensure broader coverage of relevant studies, especially those published in specialized or non-indexed journals. Additionally, future research should include multilingual studies to ensure that important contributions from non-English-speaking regions are captured. Furthermore, to complement bibliometric analyses, future research should adopt mixed-methods approaches, incorporating qualitative reviews of key studies to assess the methodological rigor, clinical applicability, and translational impact of the TyG index in cardiovascular research. This could include systematic reviews or meta-analyses of the most influential studies identified through bibliometric techniques, offering deeper insights into the utility and limitations of the TyG index.

## Conclusion

This bibliometric study offers a detailed analysis of the growing body of research on the TyG index, emphasizing its importance in metabolic and cardiovascular research. The keyword and cluster analyses revealed major research themes, including percutaneous coronary intervention, obesity indicators, arterial stiffness, heart failure, new-onset hypertension, predicting outcomes, and subclinical CAD. These findings reflect the diverse clinical applications of the TyG index, particularly in cardiovascular risk assessment and prognostic evaluation. The keyword “risk” emerged as the most frequent term, highlighting the TyG index’s central role in assessing CVD risks, alongside its utility in predicting outcomes.

Temporal and geographical analyses showed a significant increase in publications over the last decade, with a strong contribution from Asian countries, particularly China and South Korea. These trends underscore the global recognition of the TyG index as a valuable biomarker. Additionally, collaboration network analyses identified leading institutions and authors, which could facilitate further international research efforts. The citation analysis pinpointed seminal works that have shaped the understanding of the TyG index’s role in various clinical contexts, providing a foundation for future investigations.

These findings not only confirm the TyG index’s utility as a cost-effective and accessible tool for identifying insulin resistance but also highlight its emerging role in predicting outcomes related to cardiovascular and metabolic diseases. The broad spectrum of clinical applications identified in this study suggests that the TyG index could play a pivotal role in guiding therapeutic decision-making and stratifying patient risk in both resource-rich and resource-limited settings. Future research should focus on validating the TyG index’s longitudinal utility, refining its predictive accuracy through standardized cut-off values, and exploring its role in younger populations and sex-specific analyses. Additionally, its integration into existing cardiovascular risk models could enhance patient outcomes and improve global health strategies.

## Supplementary Material

## Data Availability

The datasets used and/or analyzed during the current study are accessible from the corresponding author on reasonable request.

## Research registration unique identifying number (UIN)

The current study is a bibliometric study and UIN is not applicable for this study.
