# Supplementary material for: Triglyceride-glucose index as a marker in cardiovascular diseases; a bibliometric study and visual analysis
Source: Ann Med Surg (Lond). 2025 Feb 27;87(3):1487–505. doi: 10.1097/MS9.0000000000003019 (PMC11981332; doi:10.1097/MS9.0000000000003019)
Supplement: Supplementary file 1 [file ms9-87-1487-s001.docx]

Table S1: Search formula for the databases

| Database | Search formula |
| --- | --- |
| Web of Science | (("TyG index" OR "triglyceride-glucose index" OR "triglyceride glucose index" OR "triglyceride glucose" OR "triacylglycerol glucose index") AND ("Cardiovascular Diseases" OR "Cardiovascular Disease" OR "Disease, Cardiovascular" OR "Major Adverse Cardiac Events" OR "Cardiac Events" OR "Cardiac Event" OR "Event, Cardiac" OR "Adverse Cardiac Event" OR "Adverse Cardiac Events" OR "Cardiac Event, Adverse" OR "Cardiac Events, Adverse" OR "Cardiovascular Abnormalities" OR "Abnormalities, Cardiovascular" OR "Abnormality, Cardiovascular" OR "Cardiovascular Abnormality" OR "Heart Diseases" OR "Heart Disease" OR "Cardiac Diseases" OR "Cardiac Disease" OR "Cardiac Disorders" OR "Cardiac Disorder" OR "Heart Disorders" OR "Heart Disorder" OR "Heart Failure" OR "Cardiac Failure" OR "Heart Decompensation" OR "Decompensation, Heart" OR "Heart Failure, Right-Sided" OR "Heart Failure, Right Sided" OR "Right-Sided Heart Failure" OR "Right Sided Heart Failure" OR "Myocardial Failure" OR "Congestive Heart Failure" OR "Heart Failure, Congestive" OR "Heart Failure, Left-Sided" OR "Heart Failure, Left Sided" OR "Left-Sided Heart Failure" OR "Left Sided Heart Failure" OR "Hypertension" OR "Blood Pressure, High" OR "Blood Pressures, High" OR "High Blood Pressure" OR "High Blood Pressures" OR "Myocardial Ischemia" OR "Ischemia, Myocardial" OR "Ischemias, Myocardial" OR "Myocardial Ischemias" OR "Ischemic Heart Disease" OR "Heart Disease, Ischemic" OR "Disease, Ischemic Heart" OR "Diseases, Ischemic Heart" OR "Heart Diseases, Ischemic" OR "Ischemic Heart Diseases" OR "Atherosclerosis" OR "Atheroscleroses" OR "Atherogenesis" OR "Atherogeneses" OR "Artery Disease, Coronary" OR "Artery Diseases, Coronary" OR "Coronary Artery Diseases" OR "Coronary Arteriosclerosis" OR "Arterioscleroses, Coronary" OR "Coronary Arterioscleroses" OR "Arteriosclerosis, Coronary" OR "Atherosclerosis, Coronary" OR "Atheroscleroses, Coronary" OR "Coronary Atheroscleroses" OR "Coronary Atherosclerosis" OR "Left Main Coronary Artery Disease" OR "Left Main Coronary Disease" OR "Left Main Disease" OR "Left Main Diseases" OR "Coronary Artery Disease" OR "Arrhythmia" OR "Arrythmia" OR "Cardiac Arrhythmia" OR "Cardiac Arrhythmias" OR "Cardiac Dysrhythmia" OR "Dysrhythmia, Cardiac" OR "Arrhythmias, Cardiac" OR "Atrial Fibrillations" OR "Fibrillation, Atrial" OR "Fibrillations, Atrial" OR "Auricular Fibrillation" OR "Auricular Fibrillations" OR "Fibrillation, Auricular" OR "Fibrillations, Auricular" OR "Persistent Atrial Fibrillation" OR "Atrial Fibrillation, Persistent" OR "Atrial Fibrillations, Persistent" OR "Fibrillation, Persistent Atrial" OR "Fibrillations, Persistent Atrial" OR "Persistent Atrial Fibrillations" OR "Familial Atrial Fibrillation" OR "Atrial Fibrillation, Familial" OR "Atrial Fibrillations, Familial" OR "Familial Atrial Fibrillations" OR "Fibrillation, Familial Atrial" OR "Fibrillations, Familial Atrial" OR "Paroxysmal Atrial Fibrillation" OR "Atrial Fibrillation, Paroxysmal" OR "Atrial Fibrillations, Paroxysmal" OR "Fibrillation, Paroxysmal Atrial" OR "Fibrillations, Paroxysmal Atrial" OR "Paroxysmal Atrial Fibrillations" OR "Atrial Fibrillation")) |
